# Myoimaging in the NGS era: the discovery of a novel mutation in *MYH7* in a family with distal myopathy and core-like features – a case report

**DOI:** 10.1186/s12881-016-0288-0

**Published:** 2016-03-22

**Authors:** Guja Astrea, Antonio Petrucci, Denise Cassandrini, Marco Savarese, Rosanna Trovato, Ludovico Lispi, Anna Rubegni, Manlio Giacanelli, Roberto Massa, Vincenzo Nigro, Filippo M. Santorelli

**Affiliations:** Molecular Medicine, IRCCS Stella Maris, via dei Giacinti 2, 56128 Calambrone, Pisa Italy; Center for Neuromuscular and Neurological Rare Diseases, S. Camillo-Forlanini Hospital, Rome, Italy; Department of Biochemistry, Biophysics and General Pathology (Medical Genetics), Second University of Naples, Naples, Italy; Department of Systems Medicine (Neurology), University of Tor Vergata, Rome, Italy; Telethon Institute of Genetics and Medicine, Naples, Italy

**Keywords:** Core myopathies, *MYH7*, *RYR1*, Muscle MRI, Next-generation sequencing

## Abstract

**Background:**

Myosin heavy chain 7 related myopathies are rare disorders characterized by a wide phenotypic spectrum and heterogeneous pathological features. In the present study, we performed clinical, morphological, genetic and imaging investigations in three relatives affected by autosomal dominant distal myopathy. Whilst earlier traditional Sanger investigations had pointed to the wrong gene as disease causative, next-generation sequencing allowed us to obtain the definitive molecular genetic diagnosis in the family.

**Case presentation:**

The proposita, being found to harbor a novel heterozygous mutation in the *RYR1* gene (p.Glu294Lys), was initially diagnosed with core myopathy. Subsequently, consideration of muscle magnetic resonance imaging (MRI) features and extension of family study led this diagnosis to be questioned. Use of next-generation sequencing analysis identified a novel mutation in the *MYH7*gene (p.Ser1435Pro) that segregated in the affected family members.

**Conclusions:**

This study identified a novel mutation in *MYH7* in a family where the conclusive molecular diagnosis was reached through a complicated path. This case report might raise awareness, among clinicians, of the need to interpret NGS data in combination with muscle MRI patterns so as to facilitate the pinpointing of the main molecular etiology in inherited muscle disorders.

**Electronic supplementary material:**

The online version of this article (doi:10.1186/s12881-016-0288-0) contains supplementary material, which is available to authorized users.

## Background

More than 200 different dominantly inherited mutations in *MYH7*, encoding the human slow/β myosin heavy chain, have been identified and associated with a variety of clinical myosinopathies, including hypertrophic (MIM 192600) or dilated cardiomyopathy with left ventricular non-compaction (MIM 613426), myosin storage myopathy (MIM 608358) and Laing distal myopathy (LDM) (MIM 160500) [1, 2]. Additional related clinical syndromes include congenital myopathies with features of multi-minicores (MMC) or fiber type disproportion (CFTD) in skeletal muscle, and combinations of heart and muscle involvement [3, 4]. The clinical manifestations may range from asymptomatic to limb-girdle, scapuloperoneal or distal muscle involvement [3]; congenital as well as late-onset manifestations have also been described and the clinical phenotype can vary within a single family [5].

Typical cases of LDM are characterized by early-onset ankle dorsiflexor weakness, the “hanging big toe” sign and normal or moderately elevated serum creatine kinase (CK) levels. Dropped fingers pattern of weakness, involvement of neck flexor and abdominal and facial muscles might occur [6]. As the disease progresses, variable clinical findings include quadriceps atrophy, ankle retraction, *pes cavus*, scoliosis, claw toes, a high-arched palate and myalgia. EMG shows a combination of myopathic and neurogenic features [3], and muscle biopsies can reveal (in addition to the subsarcolemmal accumulation that is a characteristic finding in myosin storage myopathy) various and unspecific findings, such as CFTD, cores or MMC, and mitochondrial abnormalities [6].

We describe the clinical and myoimaging data collected in a family with an autosomal dominant (AD) distal myopathy due to a heterozygous mutation in *MYH7*, which was initially unrecognized due to incorrect attribution of the phenotype to a variant in *RYR1*.

## Methods

The index case and her relatives underwent full neuromuscular assessment, and written informed consent for molecular genetic testing was obtained prior to genomic DNA purification from blood. Mutation screening of the proposita used an amplicon-based Sanger sequencing method focused on *RYR1* (NM_000540), as reported [7]. We also used MotorPlex [8], a targeted next-generation sequencing (NGS) panel, to investigate the coding regions of 92 genes responsible of non-syndromic muscle disorders. Variant annotation was performed as described [8] and analyses of polymorphic human variations included dbSNP (http://www.ncbi.nlm.nih.gov/projects/SNP/), EVS6500 (evs.gs.washington.edu), ClinVar (http://www.ncbi.nlm.nih.gov/clinvar/), ExAC Browser (exac.broadinstitute.org/), and 1000G dataset (www.1000genomes.org). The damaging effects of the SNVs were evaluated *in silico* using Mutation Taster (www.mutationtaster.org), PolyPhen-2 (Polymorphism Phenotyping v2) (genetics.bwh.harvard.edu/pph2/), SIFT (SIFT, http://sift.jcvi.org), and PANTHER (http://www.pantherdb.org/).

## Case presentation

Table 1 summarizes the clinical and morphological features, genetic data, and electrophysiology results of the living members in generations III and IV in this family.

**Table 1:** Summary of findings in the living members in this family

Patient	Age at onset of symptoms (y)	Age at last evaluation (y)	Severity of weakness	Contractures	Foot deformities	Scoliosis	Cardiac involvement	Disease progression	Central nuclei	Cores in type I fibers	Cores in type II fibers	Type I fibers	Increase of connective tissue	EMG
III.2	30	68	Severe distal	AT contractures	*pes cavus*	No	Yes	Slow	Yes	Yes	No	Hypo	No	Myopathic
III.3	-	66	-	-	*-*	-	-	-	-	-	-	-	-	-
III.4	35	61	Severe distal; mild proximal	AT contractures	*pes cavus*	Yes	No	Slow	Yes	Yes	No	Hypo	Yes	Myopathic
IV.1 ^a^	33	34	Moderate distal	No	*pes cavus*	No	No	Slow	-	-	-	-	NA	Myopathic
IV.2	-	27	-	-	-	-	-	-	-	-	-	-	-	-

Abbreviations: Numbers refer to patients in family tree. *y* years, *EMG* electromyography, *AT* Achille's tendon, *m* myopathic, *hypo* hypotrophic, ^a^ she did not undergo a muscle biopsy

**Fig. 1:**
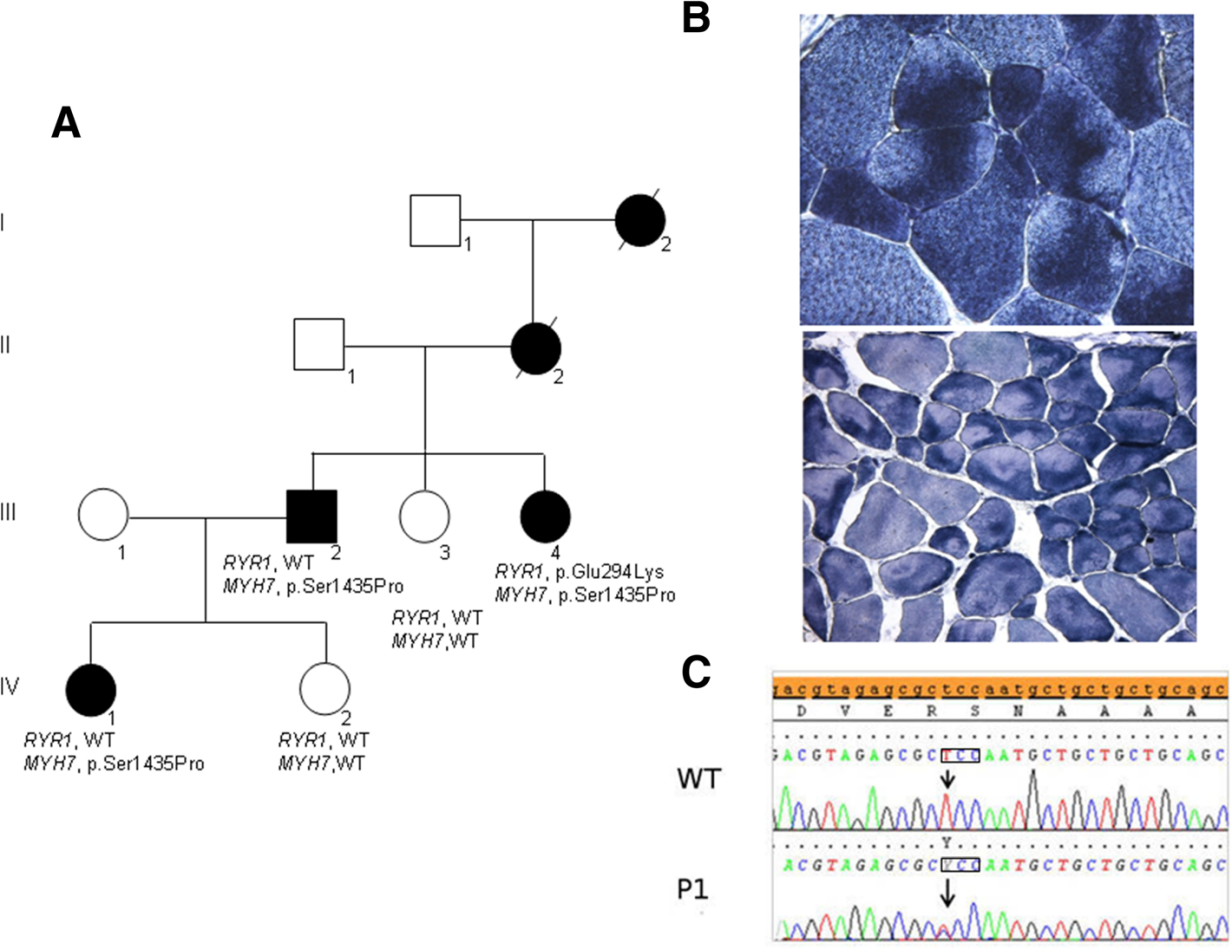
Family tree, genetic and skeletal muscle features in a patient harboring a novel mutation in *MYH7*. **a** Pedigree of the family. *Circles* are women and *squares* are men. The *RYR1* and *MYH7* genotypes of the affected individuals are also indicated. WT indicates normal sequence. **b** Muscle biopsy findings in patients III.4 (*upper panel*, final magnification x500) and III.2 (*lower panel*, x250). Light microscopy of sections stained with NADH-tetrazolium reductase showed subtle eccentric disarray of the intermyofibrillar network or eccentric cores. **c** Electropherogram of the region flanking the c.4303 T > C:p.(Ser1435Pro) mutation in the *MYH7* gene. The *arrow* indicates the nucleotide change in case III.4 (P1) when compared to a normal control (WT). The mutated codon is boxed

From the age of 30 years, the proposita (case III.4 in Fig. 1 a) showed a prevalently distal muscle phenotype characterized by stepping gait with progressive *pes cavus*, mild proximal muscle weakness in the lower limbs, and scoliosis. Upper limbs were spared. Her CK levels had always been in the normal range and her cardiac and respiratory functions were normal. At the age of 47 years, muscle biopsy did not reveal aggregates, showed fiber size variability, increased internal nuclei and histochemical evidence of eccentric “core-like lesions” [9], mainly in type I fibers (Fig. 1 b). The ATPase stains (pH 4.3, 4.6, 9.4) evidenced a slight predominance of type I fiber which were hypotrophic (see Additional file 1: Figure S1). At that time, the possibility of a core myopathy prompted us to analyze *RYR1* and we detected a heterozygous c.880G > A:p.(Glu294Lys). This mutation was predicted to be deleterious, and it was absent in human polymorphic databases, and in 200 in-house healthy Italian control chromosomes.

The family history was significant for similar muscle weakness in four relatives. Careful review of the medical notes in two patients (I.2 and II.2, both deceased) revealed that they had experienced foot drop while walking. In case III.2, a complete neurological examination and the observation of muscle biopsy findings suggestive of core myopathy suggested a disorder similar to his youngest sister. Unexpectedly, however, case III.2, did not harbor the c.880G > A:p.(Glu294Lys) in *RYR1*. Thus, the molecular diagnosis in this family remained undefined for several years until case IV.1 (the younger daughter of III.2), aged 33 years, complained of muscle weakness and sought genetic counseling. This patient displayed a less progressive muscular phenotype than her older relatives, tested negative for the mutation in *RYR1*, and refused to undergo a muscle biopsy.

**Fig. 2:**
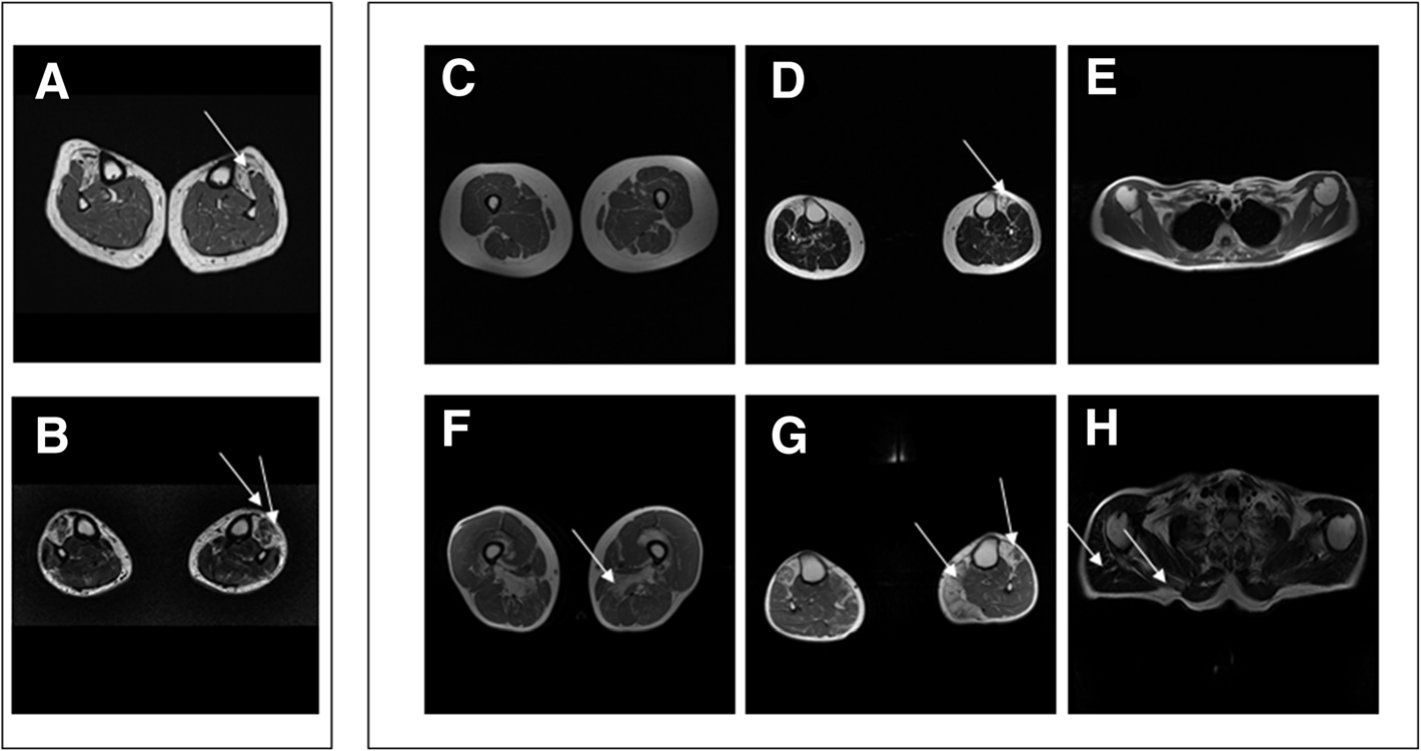
Myo-imaging in family members. Muscle MRI findings at calf level in III.4 (**a**) and III.2 (**b**) were obtained using conventional T1-weighted spin-echo transverse images. Both scans show the selective involvement of the tibialis anterior and, in III.2, of the extensor digitorium longus with sparing of gastrocnemii and soleus. Follow-up muscle MRI after 3 years in III.4 (**c**–**e**) and III.2 (**f**–**h**) obtained using conventional T1-weighted spin-echo transverse images at thigh (**c**, **f**), calf (**d**, **g**) and upper girdle (**e**–**h**) level. The findings in III.4 are almost stable whereas in III.2 it is possible to observe the additional involvement of the adductor magnus and vastus intermedius at thigh level (**f**) and of the medial gastrocnemius at calf level (**g**). The scans of the upper girdle reveal involvement of the deltoid and sternocleidomastoid muscles

To reconcile clinical and morphological findings with molecular data in this family, we asked III.2, III.4, and IV.1 to undergo muscle MRI of the thighs and calves (1.5T MR System, Signa Horizon LX, Healthcare GE, USA). Meanwhile, we used MotorPlex [8] to analyze in case III.4 a large set of genes that cause known forms of non-syndromic muscle disorders. Conventional T1-weighted spin echo images in case III.4 at the age of 61 years revealed almost complete fatty substitution of the tibialis anterior muscles with sparing of gastrocnemii and solei (Fig. 2 a), whereas there were no alterations in the thigh muscles (not shown). Likewise, muscle MRI in case III.2 at the age of 68 years documented involvement of the tibialis anterior and extensor digitorum longus (Fig. 2 b) with relative sparing of the thigh muscles (not shown). Case IV.1 showed discrete fatty degeneration of the tibialis anterior muscles. A follow-up MRI in III.2 and III.4 showed a substantially stable picture with limited additional deterioration (Fig. 2 c–h) except for the finding of involvement of the adductor magnus, vastus intermedius (Fig. 2f) and medial gastrocnemius (Fig. 2g) in III.2. In addition, neck and upper limb muscle MRI scans revealed bilateral involvement of the sternocleidomastoid and deltoid muscles in III.2 (Fig. 2h) but not in III.4.

The rather homogeneous myoimaging patterns in three affected relatives served to prioritize the >200 gene variants with a predicted deleterious significance that had emerged from the MotorPlex panel in case III.4. This strategy pinpointed only the novel heterozygous c.4303 T > C:p.(Ser1435Pro) in *MYH7* (NM_000257) (Polyphen severity score of 1.000; SIFT score 0, Damaging) as the possible causative mutation. Sanger sequencing confirmed the presence of the mutation (Fig. 1c) lying in the myosin tail domain of myosin-7 in the three living affected family members and ruled out its presence in the healthy III.3 and IV.2, and in 300 in-house Italian control chromosomes.

## Discussion

This short report provides an illustration of how a modern multidisciplinary approach can be used to obtain a correct genetic diagnosis in neuromuscular disorders and offers at least two interconnected learning points. First, it revealed an additional value of NGS tools, that is, the strategic help in revising long-term diagnoses in muscular disorders [10] as already done in other neurogenetic conditions [11]. In the index patient a variant detected in a gene associated with core myopathies (*RYR1*) was initially taken to be disease-causative, given that accepted *in silico* criteria for protein damage were met and there was morphological evidence of core myopathy in her muscle biopsy. However, since it was subsequently found that other affected family members did not carry the same mutation, we used NGS to expand the genetic analyses in combination with muscle MRI examinations to rank genetic variants. The p.Ser1435Pro in *MYH7* ― the most N-terminal mutation in the myosin-7 rod domain so far associated with LDM [6] ― finally reconciled genotype with phenotype. With hindsight, however, had the family undergone a more complete clinical assessment at the time of the initial evaluations, they could have already been diagnosed with a dominantly-inherited distal myopathy. Core-like features, the findings of fiber type disproportion in muscle biopsy, or possible insights from ultrastructural investigations as described [5], combined with weak ankle dorsiflexion should have warned on a distal phenotype related to myosin-7, as shown before [9], even prior the use of muscle MRI. Onset of a hypertrophic cardiomyopathy in one member (Table 1) was a further “red flag” that could have directed the genetic analysis towards *MYH7* and thus reduced the diagnostic “odyssey” for this family. The second learning point is related to the critical value of muscle MRI when targeted NGS is used in neuromuscular centers. Although gene panels such as MotorPlex [8] are increasingly being used in clinical practice [12], they introduce a new level of complexity, identifying multiple potential disease-associated variants that often have no clear-cut relationships with the clinical diagnosis. In the present family with a dominant transmission of core myopathy and distal muscle involvement, MRI clearly documented a preferential involvement of anterior leg muscles orienting toward a diagnosis of LDM and contributing to rank a variant in *MYH7* as disease causative.

## Conclusions

Our clinical report further corroborates how available modern tools can be used effectively in the era of clinical genomic medicine. Thus, it might raise awareness, among clinicians, of the need to interpret NGS data in combination with muscle MRI patterns so as to facilitate the pinpointing of the main molecular etiology in inherited muscle disorders.

### Ethics, consent and permissions

All the experiments were done in agreement with the Helsinki Declaration of 1975. In accordance with the rules of the ethics committee of the Center for Neuromuscular and Neurological Rare Diseases (S. Camillo-Forlanini Hospital, Rome), written informed consent was obtained from the patients for publication of this Case report and any accompanying images. A copy of the written consent is available for review by the Editor of this journal.

## Additional file

Additional file 1: Figure S1. Light microscopy of muscle sections stained with ATPASE (pH 4.3 [a], 4.6 [b], and 9.4 [c]) showing a slight predominance of type I fiber. Several type I fibers are atrophic. Hematoxylin-Eosin stain reveals muscle fiber size variability and an increased number of internal nuclei. (TIF 771 kb)

## Abbreviations

AD: Autosomal dominant
CFTD: Congenital myopathy with features of fiber type disproportion
CK: Creatine kinase
EMG: Electromyography
LDM: Laing distal myopathy
MIM: Mendelian inheritance in man
MMC: Congenital myopathy with features of multi-minicores
MRI: Magnetic resonance imaging
NGS: Next-generation sequencing

## References

[1] Tajsharghi H, Oldfors A (2013). Myosinopathies: pathology and mechanisms. Acta Neuropathol.

[2] Udd B (2007). Molecular biology of distal muscular dystrophies--sarcomeric proteins on top. Biochim Biophys Acta.

[3] Muelas N, Hackman P, Luque H, Garcés-Sánchez M, Azorín I, Suominen T, Sevilla T, Mayordomo F, Gómez L, Martí P, María Millán J, Udd B, Vílchez JJ (2010). *MYH7* gene tail mutation causing myopathic profiles beyond Laing distal myopathy. Neurology.

[4] Darin N, Tajsharghi H, Östman-Smith I, Gilljam T, Oldfors A (2007). New skeletal myopathy and cardiomyopathy associated with a missense mutation in MYH7. Neurology.

[5] Uro-Coste E, Arné-Bes MC, Pellissier JF, Richard P, Levade T, Heitz F, Figarella-Branger D, Delisle MB (2009). Striking phenotypic variability in two familial cases of myosin storage myopathy with a *MYH7* Leu1793Pro mutation. Neuromuscul Disord.

[6] Lamont PJ, Udd B, Mastaglia FL, de Visser M, Hedera P, Voit T, Bridges LR, Fabian V, Rozemuller A, Laing NG (2006). Laing early onset distal myopathy: slow myosin defect with variable abnormalities on muscle biopsy. J Neurol Neurosurg Psychiatry.

[7] Astrea G, Munteanu I, Cassandrini D, Lillis S, Trovato R, Pegoraro E, Cioni G, Mercuri E, Muntoni F, Battini R (2015). A diagnostic dilemma in a family with cystinuria type B resolved by muscle magnetic resonance. Pediatr Neurol.

[8] Savarese M, Di Fruscio G, Mutarelli M, Torella A, Magri F, Santorelli FM, Comi GP, Bruno C, Nigro V (2014). MotorPlex provides accurate variant detection across large muscle genes both in single myopathic patients and in pools of DNA samples. Acta Neuropathol Commun.

[9] Romero NB, Xie T, Malfatti E, Schaeffer U, Böhm J, Wu B, Xu F, Boucebci S, Mathis S, Neau JP, Monnier N, Fardeau M, Laporte J (2014). Autosomal dominant eccentric core disease caused by a heterozygous mutation in the MYH7 gene. J Neurol Neurosurg Psychiatry.

[10] Vasli N, Laporte J (2013). Impacts of massively parallel sequencing for genetic diagnosis of neuromuscular disorders. Acta Neuropathol.

[11] Khan TN, Klar J, Tariq M, Anjum Baig S, Malik NA, Yousaf R, Baig SM, Dahl N (2014). Evidence for autosomal recessive inheritance in SPG3A caused by homozygosity for a novel ATL1 missense mutation. Eur J Hum Genet.

[12] Ankala A, da Silva C, Gualandi F, Ferlini A, Bean LJ, Collins C, Tanner AK, Hegde MR (2015). A comprehensive genomic approach for neuromuscular diseases gives a high diagnostic yield. Ann Neurol.

