# Supplementary figures and images for: Myoimaging in the NGS era: the discovery of a novel mutation in MYH7 in a family with distal myopathy and core-like features – a case report
Source: BMC Med Genet. 2016 Mar 22;17:25. doi: 10.1186/s12881-016-0288-0 (PMC4804697; doi:10.1186/s12881-016-0288-0)

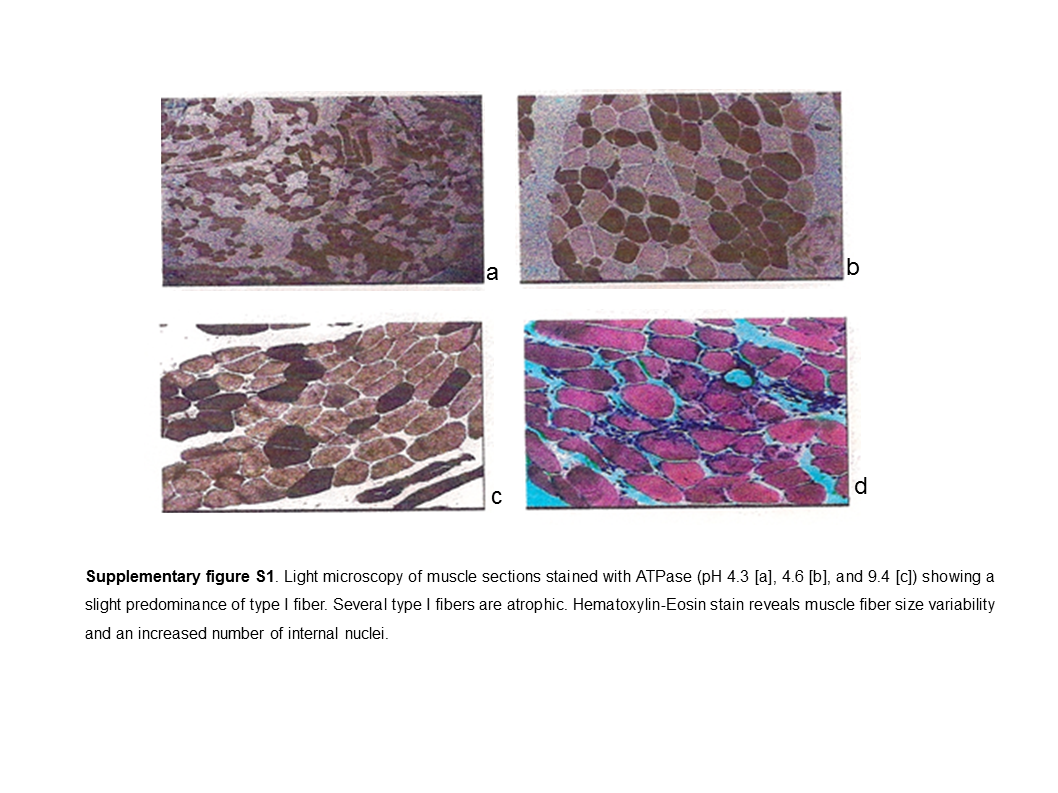

Supplement: Additional file 1: Figure S1. — Light microscopy of muscle sections stained with ATPASE (pH 4.3 [a], 4.6 [b], and 9.4 [c]) showing a slight predominance of type I fiber. Several type I fibers are atrophic. Hematoxylin-Eosin stain reveals muscle fiber size variability and an increased number of internal nuclei. (TIF 771 kb) [file 12881_2016_288_MOESM1_ESM.tif]
